# Complete mitochondrial genome of the greater Antillean parrot *Amazona ventralis* (Hispaniolan amazon)

**DOI:** 10.1080/23802359.2016.1250138

**Published:** 2016-11-11

**Authors:** Adam Dawid Urantowka, Aleksandra Kroczak, Pawel Mackiewicz

**Affiliations:** a Department of Genetics, Wroclaw University of Environmental and Life Sciences, Wroclaw, Poland;; b Department of Genomics, Faculty of Biotechnology, University of Wrocław, Wrocław, Poland

**Keywords:** *Amazona ventralis*, Hispaniolan amazon, island colonization, mitochondrial genome, parrots

## Abstract

*Androglossini* is one of the four tribes recognized within the group of neotropical parrots. The tribe includes 10 genera of which *Amazona* genus is presently represented by about 30 species. The number of species may increase in the future because paraphyly of some *Amazona* species was recently demonstrated and some subspecies were proposed to be elevated to the species rank. Evolutionary history of *Amazona* genus also remains unresolved because published phylogenies suggest contradictory scenarios concerning directions of islands-mainland colonization. Therefore, we sequenced mitogenome of *Amazona ventralis* from Greater Antilles to gain molecular data essential in future examination of this genus diversification.

Neotropical parrots, i.e. *Arinae* subfamily are divided into four tribes (Schodde et al. 2013). Tribe *Androglossini*, which grouped 10 genera including *Amazona* represented by the largest number of species distributed in Central and South America, and the Caribbean. Strong-heavy bill, short-rounded tail, prominent naked cere, and a distinct notch in the upper mandible are characteristic features for this genus. Except for Lesser Antillean (*arausiaca*, *guildingii, imperialis, versicolor*) and one continental (*vinacea*) species, their body plumage is predominantly green with variable colorations on the head, breast, wing coverts, and/or flight feathers.

Based on the variation of theses accenting colours, 27–29 *Amazona* species were primarily recognized depending on different treatments of Yellow-Headed Amazon (YHA) group, as a single (*ochrocephala*) or three species (*ochrocephala*, *auropalliata*, *oratrix*). Resurrection of the new *Alipiopsitta* genus from *Amazona xanthops* decreased the species number, which increased again with the elevation of *Amazona rhodocorytha* and *Amazona kawalli* from subspecies to species status based on genetics (Rusello & Amato 2004). *Amazona tresmariae* was distinguished from *A. oratrix* also based on results of molecular analyses (Eberhard & Bermingham 2004). The number of species may further increase because paraphyly of *auropalliata*, *ochrocephala* and *oratrix* species was recently revealed (Urantówka et al. 2014). Moreover, even *A*. *ochrocephala ochrocephala* occurred to be paraphyletic with respect to *A. aestiva* and *A. barbadensis*. Additionally, Wenner et al. (2012) suggested that two *Amazona farinosa* subspecies (*virenticeps* and *guatemalae*) should be elevated to species rank.

Evolutionary history of *Amazona* genus also remains unresolved because some clades in current phylogenies occurred biogeographically heterogeneous. Central American taxa group with South American ones, whereas Antillean species are clustered with continental taxa (Rusello & Amato 2004; Ottens-Wainright et al. 2004). So far, a clear evolutionary scenario was proposed only for continental YHA taxa and Lesser Antillean species (*arausiaca* and *versicolor*). It postulates that island parrots colonized the mainland. However, the scenario for Greater Antillean species is still controversial. Phylogenies based on CYTB gene placed Central American *Amazona albifrons* basal to all Greater Antillean species suggesting colonization of islands from mainland (Ottens-Wainright et al. 2004). However, other phylogenies based on mitochondrial and nuclear markers placed Jamaican species (*agilis*) basal to the clade with mainland and other Greater Antillean species (Rusello & Amato 2004; see also Figure 1) suggesting more complex colonizations. This indicates that more molecular data are required to reconstruct the precise *Amazona* phylogeny. So far, complete mitogenomes of only two *Amazona* species (*barbadensis* and *ochrocephala*) are available (Urantowka et al. 2013; Eberhard & Wright 2016). Therefore, we sequenced Greater Antillean *Amazona ventralis* mitogenome with 18,571 bp (GeneBank accession number KX925977) to gain appropriate molecular data for examination of *Amazona* genus diversification.

**Figure 1.**
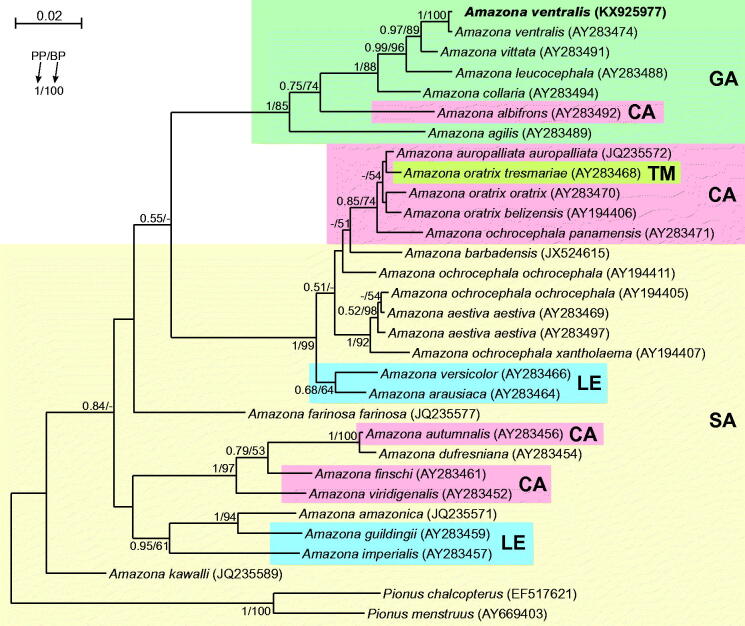
The phylogenetic tree obtained in MrBayes for *cytb* gene indicating that the studied individual (bolded) belongs to *Amazona ventralis*. The blood sample from which DNA was isolated is available in the laboratory at the Department of Genetics in Wroclaw University of Environmental and Life Sciences under the number ADUAKPM01. Clades with taxa inhabited different geographic regions were marked by various shading/colours: CA: Central America; GA: Greater Antilles; LE: Lesser Antilles; SA: South America; TM: Tres Marías Islands. An internal placement of taxa from Lesser Antilles and Central America within South American parrots implies that South America mainland was the source of those colonizations. Similarly, Tres Marías Islands were invaded from Central America, whereas some Central American parrots (e.g. *A. albifrons*) can have also Greater Antillean origin. Values at nodes, in the order shown, indicate posterior probabilities found in MrBayes (PP) and bootstrap percentages calculated in TreeFinder (BP). In the MrBayes (Ronquist et al. 2012) analysis, separate mixed substitution models were assumed for three codon positions with information about heterogeneity rate across sites as proposed by PartitionFinder (Lanfear et al. 2012). We applied two independent runs, each using four Markov chains. Trees were sampled every 100 generations for 10,000,000 generations. After obtaining the convergence, trees from the last 3,527,000 generations were collected to compute the posterior consensus. In the case of TreeFinder (Jobb et al. 2004), the separate substitution models were selected for three codon positions according to Propose Model module in this program, and 1000 replicates were assumed in the bootstrap analysis. The posterior probabilities <0.5 and bootstrap percentages <50 were omitted or marked by a dash ‘-’.

Although morphology of the analyzed specimen (Polish captive bird) was absolutely typical for *ventralis* individuals, to prove its species belonging, we compared its cytochrome b sequence with those from other *Amazona* taxa. The obtained tree (Figure 1) revealed that the analyzed individual grouped significantly with another representative of its species and this clade was sister to Puerto Rican *Amazona vittata*.
